# Corifollitropin alfa compared to daily rFSH or HP-HMG in GnRH antagonist controlled ovarian stimulation protocol for patients undergoing assisted reproduction

**DOI:** 10.5935/1518-0557.20170017

**Published:** 2017

**Authors:** Priscila Morais Galvão Souza, Bruno Ramalho de Carvalho, Hitomi Miura Nakagawa, Thalita Reis Esselin Rassi, Antônio César Paes Barbosa, Adelino Amaral Silva

**Affiliations:** 1 GENESIS - Center for Assistance in Human Reproduction, Brasília, DF, Brazil

**Keywords:** Corifollitropin alfa, gonadotropins, ovulation induction, *in vitro* fertilization, assisted reproductive technology

## Abstract

**Objective:**

This study aimed to compare the outcomes of controlled ovarian stimulation (COS) with corifollitropin alfa versus daily recombinant follicle-stimulating hormone (rRFSH) or highly purified human menopausal gonadotropin (HP-HMG) in patients undergoing *in vitro* fertilization (IVF) cycles based on gonadotropin-releasing hormone (GnRH) antagonist protocols. The primary endpoints were total number of oocytes and mature oocytes.

**Methods:**

This retrospective study looked into 132 controlled ovarian stimulation cycles from IVF or oocyte cryopreservation performed in a private human reproduction center between January 1 and December 31, 2014. Enrollment criteria: women aged < 40 years submitted to COS with corifollitropin alfa 100µg or 150µg (n = 26) and rFSH or HP-HMG in the first seven days of treatment with daily doses of 150-225 IU (n = 106); all subjects were on GnRH antagonist protocols.

**Results:**

The groups had similar mean ages and duration of stimulation. The mean number ± standard deviation of total aspirated oocytes and MII oocytes was 11.9±10 and 10.3±7.9 in the corifollitropin alfa group, and 10.9±7.2 and 8.6±5.7 in the group on rFSH or HMG (*p*>0.05). There were no significant differences in fertilization (76.9% vs. 76.8%, *p*=1.0), biochemical pregnancy (66.7% vs. 47.2%, *p*=0.1561) or embryo implantation rates (68.7% vs. 50%, *p*=0.2588) between the groups using corifollitropin alfa and rFSH or HMG, respectively.

**Conclusions:**

Corifollitropin alfa seems to be as effective as rFSH or HP-HMG when used in the first seven days of ovulation induction for patients undergoing assisted reproduction in GnRH antagonist protocols.

## INTRODUCTION

Assisted reproductive treatments often take a significant financial and emotional toll on patients, not to mention the time-consuming visits required during ovarian stimulation and the frustration inherent to a diagnosis of infertility. Standard protocols for controlled ovarian stimulation usually include daily self-administered injectable doses of gonadotropin, which increase the need for medical attention and introduce additional psychological distress as described by infertile couples; these factors combined increase the number of patients dropping out of therapy (^Rajkhowa, 2006^).

In such context, innovative strategies are needed to diminish the emotional stress caused by *in vitro* fertilization (IVF), with the development of patient-friendly, cost-effective, good quality ovarian stimulation protocols (^de Carvalho, 2016^). Decreasing the number of daily injections might mitigate the negative impact on the treated couple, improving cooperation and compliance, and maximizing results by reducing potential administration errors (^Devroey *et al.*, 2009^).

In the presence of high affinity to FSH receptors and sustained follicle-stimulating activity, it has been proven that corifollitropin alfa is able to replace the first seven daily doses of any rFSH preparation in controlled ovarian hyperstimulation prior to IVF (^Bouloux *et al.*, 2001^). Although experience with corifollitropin alfa is still incipient when compared to other gonadotropins, several studies have been carried out in recent years to assess its efficacy and compare it against traditional ovarian induction regimens (^Devroey *et al.*, 2009^; ^Mahmoud Youssef *et al.*, 2012^; ^Kolibianakis *et al.*, 2015^; ^Griesinger *et al.*, 2016^).

This study aimed to compare the response to controlled ovarian stimulation (COS) with corifollitropin alfa, and recombinant follicle-stimulating hormone (rFSH) or highly purified human menopausal gonadotropin (HP-HMG) during the first seven days in patients on GnRH antagonist protocols offered *in vitro* fertilization with intracytoplasmic sperm injection (IVF/ICSI) or oocyte cryopreservation.

## MATERIAL AND METHODS

This retrospective study included 307 COS cycles carried out between January 1 and December 31, 2014. All patients were recruited from the GENESIS Center for Assistance in Human Reproduction in Brasília, Brazil. Participants had to meet the following enrollment criteria: COS performed with either corifollitropin alfa and rFSH or HP-HMG in GnRH antagonist protocols for purposes of oocyte cryopreservation or IVF/ICSI. In the IVF/ICSI cycles, the oocytes were fertilized with sperm from the patient's partner collected from fresh semen samples; only fresh embryo transfers were considered. Cycles with oocytes submitted to preimplantation genetic diagnosis (n = 5), patients with age ≥ 40 years (n = 144), and cycles with donated oocytes (n=26) were excluded.

The treatment protocols described in the ENGAGE (^Devroey *et al.*, 2009^) and ENSURE (^Corifollitropin alfa Ensure Study Group, 2010^) trials were adopted in this study. The patients were given either a single dose of 100µg (<60kg) or 150µg (≥60kg) of corifollitropin alfa (Elonva, Schering-Plough, Brazil) or daily 200-300 IU rFSH (follitropin beta, Puregon, Schering-Plough, Brazil) on day 2 or 3 of the menstrual cycle; follitropin alfa, Gonal-f, Merck, Brazil) or daily urinary HP-HMG (menotropin, Menopur, Ferring, Brazil) was administered for the first seven days of COS, followed by daily 200-300 IU rFSH or HP-HMG in a GnRH antagonist (ganirelix, Orgalutran, Schering-Plough, Brazil or cetrorelix, Cetrotide, Merck, Brazil) regimen until final follicular maturation with human chorionic gonadotropin (hCG). The primary endpoints were the total number of oocytes and mature oocytes yielded. The secondary endpoints were fertilization, biochemical pregnancy, and implantation rates.

The Institution's Clinical Committee approved the study. Enrolled patients gave written consent to undergo assisted reproduction technology treatment and oral consent to having their data used in the study. A specific written informed consent form was not required in this study, since research data were collected exclusively from patient files.

Statistical analysis was performed on software package GraphPad Prism version 5.00 (GraphPad Software, Inc, 2007). Samples with a normal distribution were treated with the unpaired t-test; the Mann-Whitney test was used for samples with non-parametric distributions. Fisher's exact test was used in contingency analysis. The level of significance was set at *p*<0.05.

## RESULTS

A total of 132 patients were treated in our study; 26 subjects were given a single dose of corifollitropin alfa and 106 subjects were administered daily rFSH or HP-HMG for the first seven days of COS. Table 1 describes the characteristics of the patients from each of the groups.

**Table 1:** Patient characteristics per treatment group

	Corifollitropin alfa	rFSH or HP-HMG	*P*
n	26	106	
Age (years)	34.23±4.053	34.17±3.801	NS
Duration of stimulation (days)	11.92±1.896	11.87±2.168	NS

Age and duration of stimulation are expressed as means ± standard deviations; NS = not significant

The mean number of oocytes and MII oocytes was not different between the groups given corifollitropin alfa and rFSH or HP-HMG. No differences were found in terms of fertilization rates, number of transferred embryos, biochemical pregnancy rates or embryo implantation rates between patients on corifollitropin alfa and rFSH or HP-HMG (Table 2).

**Table 2:** Clinical outcomes of cycles using corifollitropin alfa, and recombinant follicle-stimulating hormone (rRFSH) or highly purified human menopausal gonadotropin (HP-HMG)

	Corifollitropin alfa	rFSH/HP-HMG	*P*^a^
Oocytes yielded, total (mean±SD)	11.99±10	10.9±7.2	NS
Oocytes yielded, MII (mean±SD)	10.3±7.9	8.6±5.7	NS
Fertilization, %	76.9	76.8	NS
Embryos transferred (mean±SD)	1.63±0,84	1.76±0.94	NS
Biochemical pregnancy, %	66.7	47.2	NS
Implantation rate, %	68.7	50	NS

NS = not significant

a Statistical analysis performed by unpaired *t*-test (normal distribution) or Mann-Whitney test (non-parametric distribution).

## DISCUSSION

New technologies have been introduced in the realm of assisted reproduction within the last two decades. Outcomes have been improved for specific groups of patients, but none of such innovations seemed to benefit the infertile population in general. Innovations in assisted reproduction have moved toward patient-friendliness and cost-effectiveness (^de Carvalho, 2016^). If corifollitropin alfa and daily gonadotropins are proven equivalent in terms of effectiveness and safety, enhancements in patient-friendliness may decrease the number of patients abandoning treatment and even turn the therapy into an attractive first choice of ovulation induction.

The ENGAGE Study was a double-blind randomized clinical trial that enrolled 1,509 women in the United States and 20 European countries to compare the use of 150µg of corifollitropin alfa during the first week of stimulation versus rFSH in daily doses of 200 IU, both in antagonist protocols. The study conducted by ^Devroey *et al.* (2009)^ showed that corifollitropin alfa and daily rFSH had a similar pregnancy rate outcome in normal responders.

In the following year, the ENSURE Study - also a randomized double-blind trial - enrolled 396 women weighing up to 60 kg submitted to ovarian stimulation for IVF using a single-dose of corifollitropin alfa 100µg or daily rFSH 150 IU for the first seven days on antagonist protocols. The study showed that corifollitropin alfa was potentially a simpler protocol for normal responders (^Corifollitropin alfa Ensure Study Group, 2010^).

Another randomized clinical trial comparing corifollitropin alfa and daily rFSH revealed that the number of oocytes yielded and pregnancy rates were similar for early or normal responders, regardless of treatment group (^Mardešič *et al.*, 2014^). However, a recent study suggested that corifollitropin alfa may lead to a greater number of retrieved oocytes and more cancelled cycles due to ovarian hyperstimulation when compared to rFSH (^Mahmoud Youssef *et al.*, 2012^).

Given the existence of adequate levels of follicular response, patient-friendliness is a relevant factor in the choice of a stimulation protocol. Women previously treated with rFSH who received corifollitropin alfa in a new cycle reported greater satisfaction with the single dose protocol, confirming that ovulation induction regimen might reduce the stress of treatment (^Requena *et al.*, 2013^). In this same study, there were no significant differences between groups in areas such as implantation rate (39.1% for corifollitropin vs. 38.4% for daily rFSH) or pregnancy rate (45.9% for corifollitropin vs. 44.4% for daily rFSH).

Our results must be considered with caution, since the biases inherent to open non-randomized retrospective studies cannot be ruled out. Although no significant differences have been reported in the literature in reproductive outcomes between follitropin alfa and beta (^Kolibianakis *et al.*, 2015^), or menotropin (^Westergaard *et al.*, 2011^), there may be differences between results in fixed and flexible GnRH antagonist regimens (^Kolibianakis *et al.*, 2003^), which were not analyzed as separate groups in our study. Moreover, variable daily gonadotropin doses used for stimulation may lead to different outcomes, especially on the number of gametes retrieved. Finally, according to a considerable number of references, GnRH agonist protocols are the first choice for women with good prognoses instead of GnRH antagonists (^Orvieto *et al.*, 2008^; ^Orvieto & Patrizio, 2013^), but data on corifollitropin alfa in GnRH agonist protocols are scarce, and impede further comparisons.

Corifollitropin alfa seems to be as effective as rFSH or HP-HMG in the first seven days of treatment for normal responders undergoing assisted reproduction cycles in a GnRH antagonist regimen.
